# Depression among inmates of Gandaki Province, Nepal: a cross-sectional study

**DOI:** 10.1186/s12888-024-05896-9

**Published:** 2024-06-14

**Authors:** Rajan Bhusal, Anjali P. C, Niraj Bhattarai, Durga Khadka Mishra, Sabina Khadka Sapkota, Shreesti Sharma, Krishna Prasad Sapkota

**Affiliations:** 1 Hospital and Rehabilitation Center for Disabled Children, Banepa, Nepal; 2 B and B Hospital, Gwarko, Nepal; 3 Manmohan Memorial Institute of Health Sciences, Kathmandu, Nepal; 4 Dean, Madan Bhandari Academy of Health Sciences, Heutada, Nepal; 5 Nepal Public Health Association, Lalitpur, Nepal; 6 Department of Sociology and Gerontology, Miami University, Oxford, OH USA

**Keywords:** Mental health, Incarcerated population, Correctional facilities, Prevalence

## Abstract

**Introduction:**

Depression is a pervasive mental health condition that affects individuals across various demographic categories, including imprisoned adults. The prevalence of mental health problems among inmates worldwide is considerably higher than in the general population, and it is estimated that 11% of inmates have significant mental disorders, such as anxiety and depression. This study aimed to find out the prevalence of depression and factors associated with it among the prisoners of Gandaki Province, Nepal.

**Methods:**

A descriptive cross-sectional study was conducted among the inmates in Gandaki Province, Nepal. Data were collected from 223 inmates, who were recruited through systematic random sampling from eight district-level prisons. The Beck Depression Inventory-II was used to measure depression, with the cumulated score dichotomized into depressed and not-depressed categories. Additionally, a structured questionnaire was employed to capture socio-demographic and imprisonment-related variables. Bivariate and multivariable logistic regressions were performed to examine the factors associated with depression.

**Results:**

Findings revealed that 18.8% of the inmates exhibited symptoms of depression. Inmates with health problems [(adjusted odds ratio (aOR) = 2.39], suicide ideation during imprisonment (aOR = 4.37), and attempted suicide before imprisonment (aOR = 7.97) had a statistically significant relationship with depression. This study revealed a notable prevalence of depression among incarcerated individuals in the Gandaki Province of Nepal.

**Conclusion:**

The findings imply a crucial need for psychosocial and rehabilitative interventions to enhance inmates’ mental health and overall well-being.

## Background

Depression is a mental health condition that changes a person’s mood, thoughts, and behaviour. It affects individuals worldwide, regardless of their socio-demographic, economic, or cultural backgrounds. According to the World Federation for Mental Health, one in 20 people worldwide experienced a depressive episode [1].In correctional systems, depression among inmates is a significant concern that demands attention and understanding. Depression is reportedly 5–10 times more prevalent among prisoners than the general population [2]. Globally, the prevalence of depression among prisoners is 37% [3], with a higher proportion observed in low- and middle-income countries (LMICs), where approximately 70% of the incarcerated population resides [4]. In Nepal, a country located in South Asia, depression among inmates is 35% [5], which is higher than the percentage among the general Nepali population, as the prevalence ranges from 4.2% [6] to 15.4% [7, 8]. Similarly, 26% of inmates in India were suffering from moderate depression and 28% from severe depression [9]. In Indonesia, 43% of the older inmates [10] and 41% of Malaysian inmates [11] also experienced significant levels of depression.

Incarcerated individuals are vulnerable to mental health problems due to the unique stressors associated with imprisonment, such as violence, overcrowding, and a lack of access to mental health services [12, 13]. These issues can exacerbate depression, anxiety, and post-traumatic stress disorder among prisoners [12]. However, prisoners face additional risk factors such as isolation, lack of social support, and harsh living conditions, which contribute to higher rates of depression due to the unique circumstances related to their incarceration [14]. Loss of personal freedom, isolation from loved ones, and the negative stigma associated with imprisonment contribute to hopelessness, despair, and depression [15]. Risk factors for depression in inmates include a family history of mental health disorders, substance abuse, a lack of social support, as well as the loss of personal freedom, isolation from loved ones, and the negative stigma associated with imprisonment itself [16]. Similarly, factors such as previous occupation, future insecurity, and social isolation in prisons [13], a history of substance abuse, and prior illnesses influence depression [17]. Additionally, a longer duration of imprisonment is associated with an increased risk of developing depression among inmates [18].

Depression among prisoners leads to heightened anxiety, stress, and even suicidal thoughts [19]. Insufficient access to mental health services within the prison system further exacerbates depressed inmates’ symptoms and makes it even more challenging for them to cope with the challenges of incarceration [20]. Studies also indicate that depression negatively impacts prisoners’ physical health [2], as prisoners with depression are likelier to have chronic health conditions, such as hypertension and diabetes [2].

The Prisons Act 2019 BS (1963 AD) of Nepal has addressed some problems concerning inmates, but dedicated policies related to mental health are lacking. However, the health provisions in the Prison Act mandate that detainees or prisoners, whether physically or mentally ill, receive medical treatment from a government doctor, with the option to seek treatment from a private doctor at their own expense. If critically ill, they must be moved to a hospital without restraints [21]. Moreover, individuals with physical issues are referred to the hospital for checkups rather than for mental health concerns. This suggests a neglect of mental health issues within prisons, with no reported mental health screening services available in Nepal [22].

Overcoming the myths and cultural barriers surrounding mental health is crucial for promoting awareness and acceptance among the Nepali population [23]. Prisoners are a neglected population, socially isolated from the community, especially in LMICs like Nepal, and research on them has been scant [5], resulting in an incomplete understanding of their situations and their challenges in prison regarding mental health [17]. Identifying the factors associated with depression among incarcerated individuals that can be modified is imperative [24]. Hence, this study aimed to find out the prevalence of depression and factors associated with it among the prisoners of Gandaki Province, Nepal.

## Methods

### Study design and setting

**Fig. 1:**
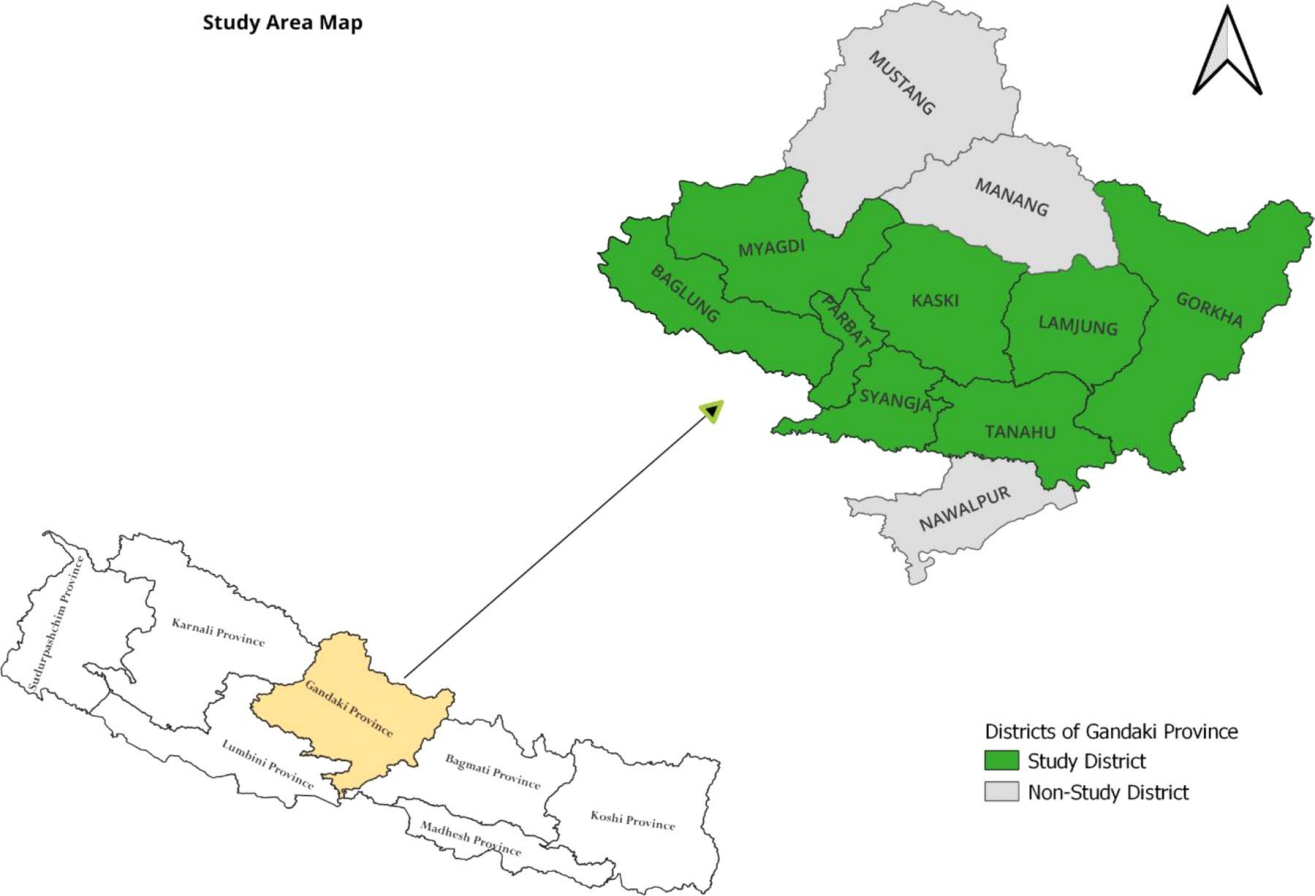
Study area map showing study districts from the Gandaki Province

A cross-sectional study was conducted in the Gandaki Province of Nepal. Gandaki Province is in the western part of the country and has 11 districts; ten districts have prisons. However, the study was conducted among eight district prisons, i.e., Baglung, Gorkha, Syangja, Tanahun, Parbat, Lamjung, Myagdi, and Kaski (Fig. 1). Only three districts—Nawalpur, Manang, and Mustang—were excluded. Nawalpur was excluded due to the absence of a prison. Manang and Mustang were excluded from the sampling process because they had few inmates, which would have skewed the representation of the overall population. Our total sampling frame comprised 1034 individuals from all districts. However, Manang and Mustang only had 2 and 4 prisoners respectively. When combined, these numbers represented less than 1% of the total population. Therefore, they were excluded from the sample size calculation as they did not adequately represent even 1% of the population. Moreover, it was not feasible to conduct data collection there due to logistics and additional cost incurred to collect data from fewer sampling population. The selection of Gandaki Province was based on the limited research available in the area. Gandaki Province covers two geographic terrains of Nepal: mountains and hills. Likewise, it also has districts of various sizes, such as districts with small towns and big cities; however, most of the districts are rural.

### Participants

A list of all eligible inmates was collected from the prison authority to construct a sampling frame for the study. The frame consisted of 1,034 inmates present at the time of the survey in all the prisons of the Gandaki Province. A proportionate sampling technique was employed to ensure a balanced sample representation. A total of 223 samples were collected based on the calculated sample size, and samples were selected proportionally from each district based on the total number of prisoners present in the prisons. Once approved and after obtaining consent, we obtained a list of inmates from the prison officer. From this list, we employed systematic random sampling, randomly selecting a starting point from a list of inmates, assigning the n^th^ number, and then choosing every n^th^ person thereafter. This approach ensured both ethical participation and unbiased selection, allowing every inmate in the prison an equal chance of being included in the study. All inmates who were mentally capable of providing informed consent were included in the study while adhering to the security protocols of the Department of Prison and District Prison Management. Prisoners who were not permitted to meet with the researchers were excluded from the study.

A final sample size of 223 inmates was calculated based on the following sample size formula n = Z²×p×(1 –p)/d², where p, the expected proportion of disease in the subject population, which was 17.6% in a similar setting [25], d is the margin of error and was set at 5%, and Z is the standard normal variate, which is 1.96 for 95% confidence interval.

### Data collection

One of the co-authors collected data through face-to-face interviews using a structured questionnaire. The questionnaire was prepared in Nepali and guided by similar research conducted in eastern Nepal [5]. The questionnaire captured depression status, socio-demographic characteristics, detention status, self-reported health problems, and prisoners’ suicide-related characteristics. The KoBo Toolbox was used to manage the questionnaire and minimize the data collection error [26]. The questionnaire was prepared in the KoBo Toolbox and loaded into a smartphone, and data were collected in April 2023.

### Measures

#### Dependent variable

Depression was assessed using Beck’s Depression Inventory second edition (BDI-II), a 21-item scale that has been validated in the context of Nepal including factor analysis, and reported a Cronbach’s alpha value of 0.88. This study also found the BDI to have a sensitivity of 0.85 and a specificity of 0.86, indicating high reliability and validity in the Nepalese context [27]. Moreover, the scale was internally reliable in measuring depression among inmates in our study, with Cronbach’s alpha value of 0.85, which is also consistent with the BDI-II validation study done in Nepal which had Cronbach’s alpha value of 0.88 [27]. A cumulated score of 21 items on the BDI scale was generated, ranging from zero to 63. The standard BDI-II tool assesses the severity of depression with scores categorized into four levels of depression: A score of 0–13 categorized as minimal depression/normal, 14–19 as mild depression, 20–28 as moderate depression, and 29–63 as severe depression [28]. Nevertheless, it has been used as a screening tool for depression globally [29]. As the 0–13 category was also categorized as a normal level for depression, we categorized the cumulated score into two categories; a score greater and equal to 14 was considered depressed, and less than 14 was not-depressed [30].

#### Independent variables

The overall health of inmates was assessed by inquiring, “Do you have any health problems?” with responses categorized as “yes” or “no.” Moreover, individuals were asked about any symptoms or discomfort they had experienced over the past three months. Similarly, suicide-related information was measured by “yes/no” questions on suicidal ideation, suicidal attempts during imprisonment, and suicidal attempts in the past before imprisonment. The duration of imprisonment was measured in months of incarceration, and participation in income-generating activities conducted by the prison encompasses the income generated from handicrafts manufactured by prisoners. Moreover, demographic characteristics such as age (in a completed year), sex, ethnicity, marital status, level of education, former occupation, and history of cigarette smoking were measured. Sex was measured as female and male. Similarly, ethnicity was recorded as *Brahmin/Chhetri*, *Dalits, Janajati*, *Madhesi*, and Muslims, but due to low observations in *Madhesi* and *Muslims*, they were merged into new groups, “ethnic minorities and Terai caste.”

Similarly, marital status was captured by four categories: Married, Separated/Divorced, Unmarried, and Widow/Widower. However, because of the low observations in some categories, Separated/ Divorced, Unmarried, and Widow/Widower were merged and created a new category of Single/Separated. Likewise, educational level was measured in three types: No formal education, School level, and Higher Education level. School-level education means grades 1–12, and Higher Education means completing a university degree or above.

### Statistical analysis

The data was extracted to MS Excel from the Kobo Toolbox and imported to SAS software version 9.4 (SAS Institute Inc, 2013. Cary, NC) for the data analysis. Descriptive and inferential statistics were calculated. A regression diagnostic was run, multicollinearity was checked before inferential statistics were run, and no multicollinearity issue was found. Multivariable logistic regression was applied to understand the associated factors for the depression of inmates.

### Ethical consideration

Ethical approval (IRC-036-079) was obtained from the Madan Bhandari Academy of Health Sciences Institutional Review Committee, Hetauda, Nepal. After reading or understanding the information sheet, all participants provided written informed consent, which included a clear and concise explanation of the study’s objectives, aims, and an overview of the potential risks and benefits associated with participation. Participants were given adequate time to review the information provided and were encouraged to seek clarification and ask questions about any aspect of the study. The study ensured that the participants’ rights and dignity were fully respected throughout the study. Participants had the right to withdraw from the interview without pressure or inducement. The confidentiality of the participants was maintained throughout the study, including during data collection, analysis, and interpretation.

## Results

This study was conducted among 223 inmates of the Gandaki Province to assess their level of depression. Table 1 depicts the socio-demographic characteristics of respondents. The mean age of incarcerated people was 36 years, and most respondents were male (71.3%). The mean months of imprisonment were 32 months with a mode of 36 months. Most inmates were Brahmin/Chhetri (42.6%), followed by Janajati (30.04%). A majority (58.7%) of the inmates had school-level education. Likewise, most inmates were married (63.2%). Almost 20% were unemployed before imprisonment, and 18% engaged in foreign employment before incarceration. Only 45.3% of the inmates reported being involved in income-generating activities inside the prison. About 39% of the inmates were convicted of sexual offences.

**Table 1:** Socio-demographic characteristics of inmates

Variables	Frequency	Percent/Mean
**Age (years)**	223	36.0 (SD = 11.9)
**Imprisoned time (months)**	223	32.3 (SD = 26.5)
**Sex**		
Male	159	71.3
Female	64	28.7
**Educational attainment**		
No formal education	60	26.9
School level	131	58.7
Higher Education	32	14.4
**Ethnicity**		
Brahmin/Chhetri	95	42.6
Janajati	67	30.1
Dalits	50	22.4
Ethnic minorities and Terai caste	11	4.9
**Marital status**		
Married	141	63.2
Single/Separated	82	36.8
**Occupation before imprisonment**		
Agriculture	26	11.7
Business	41	18.4
Foreign worker	42	18.8
Government worker	21	9.4
Student	16	7.2
Technical work	34	15.3
Unemployed	43	19.3
**Engaged in income-generating activities in prison**		
No	122	54.7
Yes	101	45.3
**Type of offence committed by respondents**		
Drug-related	33	14.8
Property	16	7.2
Sexual offence	87	39.0
Violence	21	9.4
Others	66	29.6

Table 2 depicts the health-related characteristics of incarcerated adults. More than 34% of inmates reported that they have health problems. Moreover, 58.3% smoked before imprisonment. Thirteen per cent of inmates in this study reported suicidal ideation inside the prison, and 7.27% of the total inmates had attempted suicide in prison. Likewise, 4.0% of inmates had also attempted suicide before the imprisonment.

**Table 2:** Health-related characteristics of the inmates

Variables	Frequency	Percent
Have health problem		
Yes	76	34.1
No	147	65.9
**Smoking history before imprisonment**		
Yes	130	58.3
No	93	41.7
**Suicide ideation during imprisonment**		
Yes	29	13.0
No	194	87.0
**Attempted suicide during imprisonment**		
Yes	16	7.2
No	207	92.8
**Attempted suicide before imprisonment**		
Yes	9	4.0
No	214	96.0

Table 3 presents the statistics of depression among inmates. In this study, it was found that 18.8% of the inmates were depressed.

**Table 3:** Prevalence of depression among inmates

Depression status	Frequency	Percent
Depressed	42	18.8
Not Depressed	181	81.2

Table 4 displays the unadjusted and adjusted odds ratios (aOR) with 95% confidence intervals (CI) for the association of depression of inmates with each variable analyzed. Depression was significantly associated with health problems, suicidal ideation during imprisonment, and attempted suicide before imprisonment. The results show that inmates who reported having health problems had significantly higher odds of depression (aOR = 2.39; 95% CI = 1.01–5.66) when controlling for other variables.

Similarly, inmates who reported suicidal ideation during imprisonment had significantly higher odds of depression (aOR = 4.37; 95% CI = 1.04–18.30), while those who attempted suicide during imprisonment did not show a statistically significant association with depression. Nevertheless, inmates who reported attempting suicide before imprisonment had significantly higher odds of depression (aOR = 7.97; 95% CI = 1.43–44.46). However, the association between socio-demographic characteristics and depression was not statistically significant.

**Table 4:** Factors associated with depression among inmates

Variables	Unadjusted OR (95% CI)	Adjusted OR (95% CI)
**Have health problem** (Ref = No)		
Yes	2.29 [1.16–4.53]^*^	2.39 [1.01–5.66]^*^
**Smoking history before imprisonment** (Ref = No)		
Yes	0.36 [0.18–0.72]^**^	0.45 [0.18–1.15]
**Suicide ideation during imprisonment** (Ref = No)		
Yes	4.63 [2.01–10.62] ^***^	4.37 [1.04–18.30]^*^
**Attempted suicide during imprisonment** (Ref = No)		
Yes	2.85 [0.97–8.34]	0.88 [0.14–5.61]
**Attempted suicide before imprisonment** (Ref = No)		
Yes	5.98 [1.53–23.33] ^*^	7.97 [1.43–44.46] ^*^
**Engaged in Income Generating Activities in Prison** (Ref = No)		
Yes	0.61 [0.31–1.23]	0.82 [0.34–1.96]
**Offence type (Ref = Violence)**		
Drug-related	2.76 [0.29–26.54]	4.40 [0.35–56.02]
Property	4.61 [0.43–49.29]	2.62 [0.18–38.38]
Sexual offence	3.51 [0.43–28.49]	4.56 [0.47–43.37]
Others	9.33 [1.17–74.26]^*^	5.67 [0.59–54.49]
**Imprisoned time**	0.98 [0.97–1.00]	0.98 [0.96–1.00]
**Age of respondents**	1.02 [0.99–1.04]	1.01 [0.98–1.05]
**Sex** (Ref = Male)		
Female	3.21 [1.60–6.43]^*^	1.27 [0.39–4.10]
**Education** (Ref = No formal education)		
Higher Education	0.43 [0.14–1.30]	0.64 [0.14–3.02]
School level	0.40 [0.19–0.83]^*^	0.43 [0.15–1.23]
**Ethnicity** (Ref = Brahmin/Chhetri)		
Janajati	0.75 [0.32–1.75]	0.98 [0.35–2.75]
Dalits	1.21 [0.52–2.80]	0.79 [0.22–2.78]
Ethnic minorities and Terai caste	1.60 [0.39–6.65]	1.81 [0.30–10.89]
**Marital status** (Ref = Single/Separated)		
Married	0.73 [0.37–1.44]	0.65 [0.27–1.57]
**Occupation before imprisonment** (Ref = Unemployed)		
Agriculture	2.00 [0.67–5.96]	2.12 [0.52–8.63]
Business	0.65 [0.21–2.02]	0.71 [0.17–2.98]
Foreign worker	0.76 [0.25–2.26]	0.84 [0.20–3.49]
Government worker	1.18 [0.34–4.10]	1.45 [0.27–7.66]
Student	0.87 [0.20–3.73]	0.80 [0.12–5.33]
Technical work	0.37 [0.09–1.47]	0.42 [0.07–2.52]

* p-value < 0.05; ** p-value < 0.01; *** p-value < 0.001;

95%CI = 95% Confidence Interval; OR = Odds Ratio

## Discussion

Our study, conducted to determine the prevalence of depression among inmates and the associated factors, revealed that 18.8% of the incarcerated individuals reported experiencing depression, as measured by the self-reported BDI-II screening tool. Moreover, having health problems, suicide attempts before imprisonment, and suicide ideation during imprisonment were associated with depression.

Similar results on the prevalence of depression as of our study were found in Nigeria, ranging from 14.8% [31] to 20.8% [14], and in India, with 25.7% [18]. However, the prevalence of depression among inmates in Gandaki Province was higher than the study conducted in France at 8.7% [32]. In contrast, our study’s result was lower than other studies done in Nepal, as 35.3% and 45.6% of the inmates had depression in eastern [5] and Dilibazar central Nepal [17], respectively. Similarly, other LMICs reported higher rates of depression among inmates, e.g., Ethiopia with 43.8% [33] and Malaysia with 40.7% [34]. Moreover, a meta-analysis conducted in 2020 suggested that the pooled prevalence of depression among inmates was 36.9%, and the prevalence was 19.1% when depression was measured with diagnostic tools [3]. The latter result is similar to ours; however, BDI-II is a depression screening tool, not a diagnostic, but is used prominently in categorizing the severity of depression. The differences in results among various countries could be due to different instruments for assessing depression and study settings. Similarly, the study design adopted also makes differences in reporting depression, and usually, cross-sectional studies report lower prevalence [3].

The difference in depression prevalence could be attributed to the varying geographic locations, socio-economic factors of inmates, and prison conditions [34]. Also, research has found that inmates could exaggerate the illness so that they could leave the prison, which is called malingering. The malingering may have exaggerated the depression among inmates studies [28]. Moreover, measurement tools such as BDI-II and the Patient Health Questionnaire (PHQ-9) introduce variability in prevalence rates due to differences in sensitivity and specificity. The PHQ-9 has a sensitivity and specificity of 88% [35] whereas BDI-II has 85% and 86% [27]. The patterns of questions asked in these tools also play a role in observing diverse prevalence rates of depression [36].

This study found a significant association between having health problems and depression. However, a study conducted in eastern Nepal did not find any statistically significant association between self-rated health and depression of inmates when controlled for other variables, although the relationship was significant in bivariate analysis [5]. Physical health declines may be associated with loss of functioning, feelings of hopelessness and helplessness, chronic pain, identity threat, and a need for increased social support, which can be challenging to obtain in incarceration. These factors may contribute to depression in inmate populations [37]. In our study, more than one-third of the inmates had self-reported health problems, and almost two-thirds were smoking in the past, which suggests that incarcerated adults are at peril of physical health issues and ultimately at risk of depression.

Furthermore, the study found a significant association between suicidal ideation during imprisonment and depression, consistent with the study conducted in eastern Nepal [5] and the USA [38]. Suicide ideation among inmates during imprisonment can be influenced by factors such as psychological distress, lack of social support, traumatic experiences, lengthy sentences and loss of hope, overcrowding, violence, and a history of previous suicide attempts [19, 39]. The prison environment, with its harsh conditions and separation from support networks, can contribute to feelings of hopelessness and isolation [40]. Additionally, pre-existing trauma, substance abuse, and a perceived lack of rehabilitation prospects can intensify the risk of suicide ideation [41]. Addressing these factors through comprehensive mental health support is crucial to prevent suicide ideation and promote rehabilitation [41].

Similarly, attempted suicide before imprisonment was found to be significantly associated with depression, which coincides with a previous study conducted among vulnerable prisoners and a separate study involving Northern Irish prisoners, which yielded similar results, indicating a strong association between prior suicide attempts before incarceration and depression [42]. Additionally, prisoners with a history of self-harm are more prone to exhibiting various depressive symptoms compared to their incarcerated counterparts without such a history [43]. This suggests an enduring susceptibility to self-harm and potentially suicidal tendencies among these individuals [42, 44]. This may be because individuals who have previously committed self-harm and/or exhibited suicidal behaviours may be particularly unable to cope with these initial stresses and be more likely to feel hopeless [45].

Meanwhile, socio-demographic characteristics, such as age, sex, marital status, education, ethnicity, occupation, and imprisoned time, were not associated with depression among the inmates. Similarly, a study in the regional prison of eastern Nepal [4] did not find any association with socio-demographic variables. However, a study conducted in India, and Western Ethiopia found that the age of the prisoners was associated with depression [18, 46, 47]. Inmates can have different levels of resilience and unique personality traits that make them different from individuals who have not committed crimes [48]. It is important to note that their criminal personality often has a stronger impact on their actions than their background or demographic characteristics before they commit the crime [48].

### Strengths and limitations of the study

The study has several strengths and limitations. One of the key strengths of the study is its focus on an understudied population, highlighting the mental health concerns of inmates in Nepal. The study respondents were the representative of the Gandaki Province. Additionally, the study utilized a standardized measure for assessing depression, providing a reliable and valid measure of the participant’s depressive symptoms. However, the study also has several limitations. Although samples were representative of the whole province, it was a relatively small sample size, which caused the confidence interval to become wider.

This study is limited by self-reported data, which can be susceptible to recall bias. Inmates might be hesitant to admit to intentionally manipulating the system or forgetting instances of such behaviour. Additionally, malingering, where individuals exaggerate symptoms for secondary gain, is a documented concern in depression research among prisoners [28] and some respondents could have done that.

In addition, we could not check for the reverse causality on the association of suicidal ideation, attempt, and depression. We believe that depression could also cause suicidal ideation. Future studies could benefit from larger sample sizes and the inclusion of objective measures to supplement self-reported data. Moreover, extensive research is necessary to gain an in-depth understanding of the underlying factors contributing to depression in an incarcerated population. Despite these limitations, the study provides valuable insights into the mental health of inmates in Nepal, highlighting the need for interventions to address depression and other mental health concerns in this population.

### Study implications

The substantial prevalence of depression (18.8%) and suicidal ideation during imprisonment (13.0%) implies that inmates are vulnerable to mental health issues in the prisons. The strong association between pre-existing health issues, suicidal behaviour prior to incarceration, and depression suggests that many inmates enter prison with underlying mental health issues. These findings highlight the need for mental health screening, diagnosis, and treatment, along with suicide prevention interventions in prisons. Moreover, the study’s implications could be linked to the rehabilitation of inmates after their release from prison. The general population of Nepal has limited awareness of prisoners’ mental health, and social and cultural barriers hinder open discussions about mental health issues [49, 50]. As a result, released prisoners are less likely to seek mental health support and are more prone to re-offend [51]. Also, depression reduces inmates’ motivation for rehabilitation, affecting their participation in reintegration programs [39]. Hence, mental health promotion and prevention interventions targeted at inmates in prison have long-term effects after their release and resettlement in the community.

## Conclusion

Our findings revealed that 18.8% of incarcerated individuals reported experiencing depression. Furthermore, our analysis indicated significant associations between depression and various factors, including the presence of health problems, history of suicide attempts prior to incarceration, and occurrence of suicide ideation during imprisonment. The prevalence of depression among inmates highlights the importance of addressing their mental health concerns to ensure their overall well-being and successful rehabilitation. Implementing targeted interventions and support systems within correctional facilities can alleviate the burden of depression and facilitate effective reintegration into society.

## Data Availability

The datasets used and/or analysed during the current study available from the corresponding author on reasonable request.
